# Supplementary material for: Novel Ran-RCC1 Inhibitory Peptide-Loaded Nanoparticles Have Anti-Cancer Efficacy In Vitro and In Vivo
Source: Cancers (Basel). 2019 Feb 14;11(2):222. doi: 10.3390/cancers11020222 (PMC6406988; doi:10.3390/cancers11020222)
Supplement: Supplementary file 1 [file cancers-11-00222-s001.pdf]

Supplementary Materials: Novel Ran-RCC1 Inhibitory Peptide-Loaded Nanoparticles Have Anti-Cancer Efficacy in Vitro and in Vivo

Yusuf A. Haggag, Kyle B. Matchett, Robert A. Falconer, Mohammad Isreb, Jason Jones, Ahmed Faheem, Paul McCarron and Mohamed El-Tanani

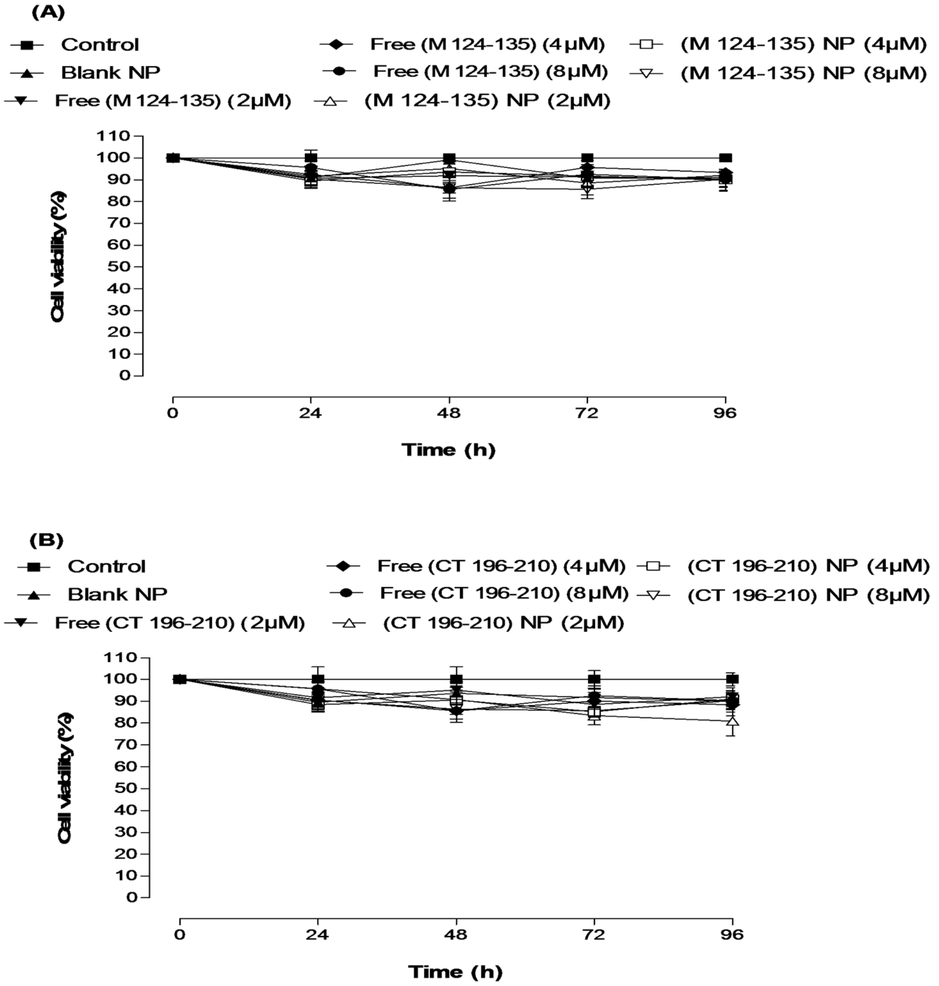

Figure S1. MDA-MB-231 cell viability results of different doses of M 124–135 peptide and M 124–135 peptide-loaded NP (**A**) and CT 196–210 peptide and CT 196–210 peptide-loaded NP (**B**) after 24, 48, 72 and 96 h.

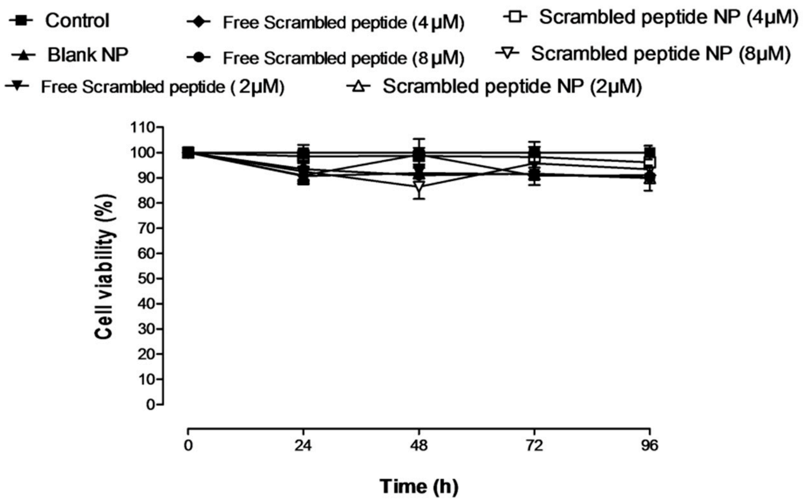

Figure S2. MDA-MB-231 cell viability results of different doses of scramble peptide and scramble peptide-loaded NP after 24, 48, 72 and 96 h.

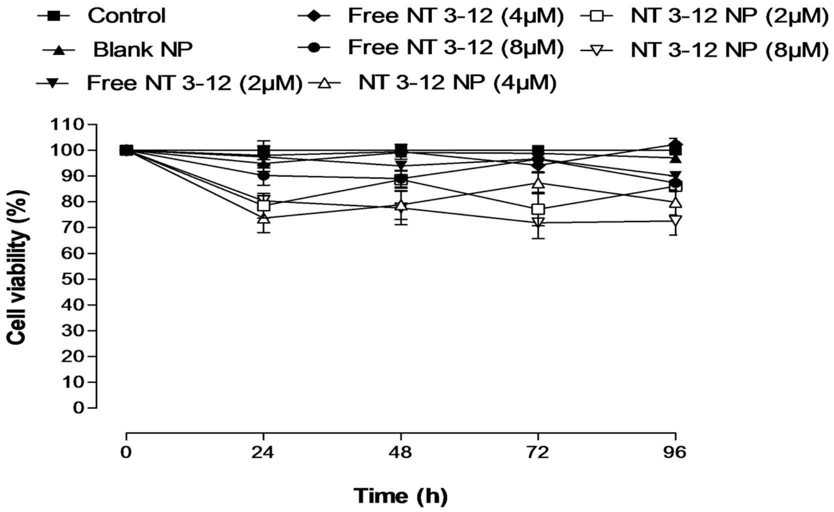

Figure S3. MCF-10a cell viability results of different doses of NT 3–12 peptide and NT 3–12 peptide-loaded NP after 24, 48, 72 and 96 h.

© 2019 by the authors. Licensee MDPI, Basel, Switzerland. This article is an open access article distributed under the terms and conditions of the Creative Commons Attribution (CC BY) license (http://creativecommons.org/licenses/by/4.0/).
